# Protection Against Post-resuscitation Acute Kidney Injury by N-Acetylcysteine via Activation of the Nrf2/HO-1 Pathway

**DOI:** 10.3389/fmed.2022.848491

**Published:** 2022-05-17

**Authors:** Shiwei Wang, Guoxiang Liu, Tianyuan Jia, Changsheng Wang, Xiaoye Lu, Lei Tian, Qian Yang, Changqing Zhu

**Affiliations:** Department of Emergency Medicine, Renji Hospital, School of Medicine, Shanghai Jiaotong University, Shanghai, China

**Keywords:** cardiac arrest, cardiopulmonary resuscitation, acute kidney injury, nuclear factor erythroid2-related factor2, haem oxygenase-1, N-acetylcysteine

## Abstract

**Background and Objective:**

Acute kidney injury (AKI), the common complication after cardiopulmonary resuscitation (CPR), seriously affects the prognosis of cardiac arrest (CA) patients. However, there are limited studies on post-resuscitation AKI. In addition, it has been demonstrated that N-acetylcysteine (N-AC) as an ROS scavenger, has multiorgan-protective effects on systemic and regional ischaemia-reperfusion injuries. However, no studies have reported its protective effects against post-resuscitation AKI and potential mechanisms. This study aimed to clarify the protective effects of N-AC on post-resuscitation AKI and investigate whether its potential mechanism was mediated by activating Nrf-2/HO-1 pathway in the kidney.

**Methods:**

We established cardiac arrest models in rats. All animals were divided into four groups: the sham, control, N-AC, and ZnPP groups. Animals in each group except for the ZnPP group were assigned into two subgroups based on the survival time: 6 and 48 h. The rats in the control, N-AC, and ZnPP groups underwent induction of ventricular fibrillation (VF), 8 min untreated VF and cardiopulmonary resuscitation. Renal function indicators, were detected using commercial kits. Renal pathologic changes were assessed by haematoxylin–eosin (HE) staining. Oxidative stress and inflammatory responses were measured using the corresponding indicators. Apoptosis was evaluated using terminal uridine nick-end labeling (TUNEL) staining, and expression of proteins associated with apoptosis and the Nrf-2/HO-1 pathway was measured by western blotting.

**Results:**

N-AC inhibited post-resuscitation AKI. We observed that N-AC reduced the levels of biomarkers of renal function derangement; improved renal pathological changes; and suppressed apoptosis, oxidative stress, and inflammatory response. Additionally, the production of ROS in the kidneys markedly decreased by N-AC. More importantly, compared with the control group, N-AC further upregulated the expression of nuclear Nrf2 and endogenous HO-1 in N-AC group. However, N-AC-determined protective effects on post-resuscitation AKI were markedly reversed after pretreatment of the HO-1 inhibitor zinc protoporphyrin (ZnPP).

**Conclusions:**

N-AC alleviated renal dysfunction and prolonged survival in animal models of CA. N-AC partially exerts beneficial renal protection via activation of the Nrf-2/HO-1 pathway. Altogether, all these findings indicated that N-AC as a common clinical agent, may have the potentially clinical utility to improve patients the outcomes in cardiac arrest.

## Introduction

Recently, recovery rates of spontaneous circulation after cardiac arrest (CA) have markedly improved; however, survival rates are still significantly low, with the survival rates for Asia, Europe, and America being 5.4, 8, and 11.7%, respectively (1–3). Following successful cardiopulmonary resuscitation (CPR), systemic ischaemia/reperfusion (I/R) injury can occur after return of spontaneous circulation (ROSC). This may lead to post-cardiac arrest syndrome (PCAS) resulting in increased morbidity and mortality rates, and poor prognosis (4, 5).

In the past decades, there are limited studies on post-resuscitation acute kidney injury (AKI). However, AKI is a common complication and a characteristic feature of PCAS. In out-of-hospital post-cardiac arrest patients, AKI prevalence was 48%, and the occurrence of AKI was strongly associated with the day-30 mortality (6). Therefore, alleviating post-resuscitation AKI might be a potential therapeutic strategy to delay the progression of PCAS and improve outcomes of CA patients. Post-resuscitation AKI is triggered by systemic I/R injury (7), which occurs when blood flow is restored and reactive oxygen species (ROS) production is induced (8). The accumulation of abnormal ROS triggers a cascade of reactions including inflammatory response, oxidative stress, and cell apoptosis, which results in renal I/R injury (9). Activation of the endogenous antioxidant system reduces ROS production thereby protecting the kidneys from I/R injury (8, 10, 11). Hence, the stimulation of antioxidative systems is a potential method for the treatment of post-resuscitation AKI.

Nuclear factor erythroid2-related factor2 (Nrf2), a redox-sensitive transcription factor, has been recognized as a critical hub in the antioxidative system that inhibits oxidative stress during renal I/R injury (12). Generally, Nrf2 is kept inactive bound to cytoplasmic Kelch-like ECH-associated protein-1 (Keap-1) (13). When Nrf2 is activated under oxidative stress, it dissociates from keap1, translocates into the nucleus, and then interacts with the antioxidant response element (ARE) (9). Furthermore, activated Nrf2 upregulates multiple genes transcription, including haem oxygenase-1 (HO-1) superoxide dismutase (SOD), glutathione peroxidase (GSH-PX), quinone oxidoreductase-1 (NQO1) (14). HO-1 is a vital enzyme that protects multiple organs from I/R injury by exerting anti-inflammatory, anti-oxidative, and anti-apoptotic effects (15–17). Moreover, the Nrf2/HO-1 pathway is participated in a cascade of repair processes in renal diseases, containing renal I/R injury (18–20). Therefore, Nrf2/HO-1 antioxidative pathway activation may be a promising therapeutic target on post-resuscitation AKI.

N-acetylcysteine (N-AC) is an ROS scavenger, which has multiple beneficial effects (21), such as antioxidative, anti-inflammatory, and anti-apoptosis (22–24). N-AC's notable successful clinical application have been verified in substantial diseases, certainly, including various renal disease. Studies have shown that N-AC has therapeutic effects on AKI in multiple experimental animal models of sepsis, haemorrhagic shock, asphyxiated newborn model and renal I/R injury (25–28). In the renal I/R injury model, the protective mechanism of N-AC is mediated by its specific activation of the Nrf2-dependent cytoprotective pathway, which then produces anti-oxidative and anti-apoptotic effects, eventually alleviating kidney injury (29). However, the effects of N-AC on post-resuscitation AKI remain unknown.

Here, we demonstrated the effects of N-AC on post-resuscitation AKI and survival duration in rat models of CA and investigated the potential mechanism through which these effects are mediated. We hypothesized that N-AC has protective effects against post-resuscitation AKI and improves outcomes by activating the Nrf2/HO-1 antioxidative pathway.

## Materials and Methods

### Animals

Eighty healthy male Sprague-Dawley rats (age, 15–16 weeks; body weight, 500–600 g) were obtained from Shanghai SLAC Laboratory Animal. All animals were provided unlimited access to food and water. They were kept under controlled conditions, with room temperature maintained at 22 ± 1°C and a 12 h light-dark cycle for 2 week prior to surgery. All procedures were performed according to the ARRIVE guidelines and relevant official guidelines under the approval of the Animal Care and Use Committee of Ren Ji Hospital, School of Medicine, Shanghai Jiao Tong University. All surgical interventions were performed under pentobarbital sodium anesthesia to avoid suffering.

### Animal Preparation

This animal experiment was performed according to the Utstein-style guidelines, which uniformly reported laboratory CPR research (30). All animals were food-fasted overnight, but with normal access to water. Animals were anesthetized via intraperitoneal injection of pentobarbital (45 mg/kg), and additional doses were injected every hour to maintain anesthesia. Endotracheal intubation was performed using 14 G catheters (Abbocath-T, USA). Two fluid-filled PE-50 (Becton-Dickinson, Franklin Lakes, NJ, USA) catheters were advanced: one from the femoral artery into the descending aorta for haemodynamic monitoring, and the other into the femoral vein to deliver the corresponding drugs. An intravenous catheter (BD Insyte™, USA, 18 GA 1.75 IN) was placed into the right ventricle through the right external jugular vein. The preserved pacing guidewire was inserted into the right ventricle from the intravenous catheter to induce ventricular fibrillation (VF). All catheters were coated with heparinized saline solution (2.5 IU ml 1). The body temperature was monitored using a rectal thermometer. During surgery, the rectal temperature was maintained at 37 ± 0.5°C using an electric heating plate of pet (K&B, Shandong, China) and lamp. Subcutaneous needle electrodes were inserted in the legs for continuous recording of the electrocardiogram (ECG).

### Experimental Protocol

After animal baseline information was recorded, animals were divided into four groups: the sham, control, N-AC and ZnPP groups (*n* = 10, 30, 30, 10, respectively). Animals in each group except for the ZnPP group were assigned into two subgroups based on the survival time: 6 and 48 h. Animals in the control and sham groups underwent surgery with and without VF induction, respectively, and both were administered solvent mixtures intravenously (composed of physiological saline and DMSO) at 5 min after ROSC. Animals in the N-AC and ZnPP groups were administered 150 mg/kg N-AC via intravenous injection at 5 min after ROSC and zinc protoporphyrin (ZnPP, 20 mg/kg, a specific inhibitor of HO-1, dissolved in DMSO) was intraperitoneally injected 24 h prior to CA, respectively.

Before inducing VF, researchers obtained baseline measurements and established mechanical ventilation. For the animal ventilator, the specific parameters were set to a respiratory rate of 100 breaths/min, tidal volume of 6 ml/kg, fraction of inspired oxygen (FiO2) of 0.21, and aspiration ratio of 1:2. VF was induced by an electric stimulation machine, which implemented direct and stable electric stimulation with a frequency of 60 Hz and a strength of 2.5 mA. The current flow, delivered to the right ventricle, was sustained for 2.5 min to guard against spontaneous defibrillation. Mechanical ventilation was discontinued and disconnected with the beginning that VF induced. Cardiac arrest was confirmed by a mean arterial pressure (MAP) <20 mmHg maintained for at least 3 min, and the VF waveform on the ECG monitor. After 8 min of unsettled VF, advanced life support was started, the animals were ventilated at a respiratory rate of 100 breaths/min, FiO2 of 100%, and mechanical precordial compression was initiated by an electric-actuated device. Chest compression was maintained at a rate of 200 beats/min by changing the current intensity. The compression depth was adjusted to 1–1.5 cm to maintain the MAP above 20 mmHg by adjusting flexible and adjustable mechanical lever. In addition, in the period of chest compression, the animal position was further secured by tapes to ensure a correct, constant and stable place for compression on the chest. Adrenaline (0.02 mg/ml) was administered to the animals at the beginning of CPR and repeated as needed. After chest compression of 3 min, defibrillation was delivered with a 3-J biphasic waveform shock to achieve ROSC. ROSC was defined as the restoration of sinus rhythm with a MAP >60 mmHg lasting at least 5 min. If ROSC was not achieved, chest compression was immediately restarted, and defibrillation was delivered every 30 s. And this cycle was repeated a maximum of 3 times. If sustained ROSC was not restored in three cycles, CPR was considered to have failed, and the animals were excluded from this study. After successful resuscitation, animals in each group except for the ZnPP group were assigned into two subgroups based on the survival time: ROSC 6 h and ROSC 48 h. Blood samples were taken at baseline, 2, 4, and 6 h after ROSC in the ROSC 6 h subgroup. The animals were then euthanised with an overdose of pentobarbital and their kidneys were removed immediately at 6 h after ROSC for subsequent tests. All animals in the ZnPP group underwent euthanasia at 6 h after ROSC. 6 h after ROSC, all catheters were removed in the ROSC 48 h subgroup, the animals were transferred back to their cages and were closely observed for 48 h after ROSC. Additionally, blood samples were obtained from the caudal veins of this subgroup at 24 h after ROSC. If the animals died before 48 h after ROSC, the living time after ROSC was recorded.

### Assessment of Renal Function

Blood samples were obtained at the stipulated time points: at baseline and 2, 4, 6, 24, and 48 h, respectively after ROSC. The samples were centrifuged at 3,000 rpm for 10 min and the supernatants were collected in EP tubes and stored at −80°C until analysis. The biomarkers of renal injury, including neutrophil gelatinase-associated lipocalin (NGAL), serum creatinine (Scr), and blood urea nitrogen (BUN), were determined with commercial enzyme-linked immunosorbent assay (ELISA) kits (Multi Sciences, Hangzhou, China) in accordance with the manufacturer's instructions.

### Histopathological Examination of Kidneys

At 6 and 48 h after ROSC, the rats were euthanised, and their left kidneys were fixed in 4% paraformaldehyde. The renal tissues were then dehydrated, cleared, embedded in paraffin, and cut into 4 μm sections. The prepared sections were stained with haematoxylin and eosin (H&E). HE-stained sections were observed to assess pathological changes in the renal tissues under an optical microscope. The structural damage to the kidney was evaluated by histological analysis according to the tubular injury score (TIS). For each section, we randomly captured 10 non-overlapping high-power fields (400 × magnification). Tubular injuries included the loss of normal tubular architecture and brush border, and the formation of intratubular casts. TIS was quantified by the percentage of damaged tubules, and was defined as follows: grade 0, no damage; grade 1, <25%; grade 2, 25–49%; grade 3, 50–74%; and grade 4, ≥75%.

### Terminal Uridine Nick-End Labeling (TUNEL) Staining

The renal tissues were preserved in 4% paraformaldehyde, embedded in paraffin blocks, and cut into 10 μm sections. To investigate apoptosis, terminal deoxynucleotidyl transferase d-UTP nick end labeling (TUNEL) assay was performed by using a TUNEL staining kit (Servicebio, Wuhan, China) in adherence to the manufacturer's instructions. After treatment with the assay kit, the sections were observed under a fluorescence microscope and images were collected for quantification using Image J. This kit was labeled with CY3 fluorescein and the positive apoptotic nuclei were red.

### Detection of ROS Generation

The production of total ROS in renal tissues was measured by dihydroethidium staining (ROS fluorescent probe; SIGMA, Shanghai, China) according to the manufacturer's protocol. Kidney tissues were briefly embedded in OCT medium and cut into cryosections (10 μm). After treatment with ROS staining solution, cryosections were washed with PBS solution and then incubated with DAPI solution at room temperature for 10 min and kept in the dark. The prepared cryosections were observed and imaged using an ortho-fluorescent microscope (NIKON ECLIPSE C1, Tokyo, JAPAN). According to the protocol, the nucleus, labeled with DAPI stains blue, and ROS-positive cells labeled with fluorescein stain red. Renal sections that stained positive for ROS were evaluated and compared among the groups.

### Determination of Inflammatory Factors Level

For the measurement of inflammatory cytokines, blood samples were collected at different time points and immediately centrifuged at 12,000 rpm for 10 min. Then, the supernatants were stored at −80°C until analysis. Serum levels of inflammatory cytokines (IL-6, IL-10, and TNF-α) were quantified by ELISA using a commercial kit (Multi Sciences, Hangzhou, China). Absorbance was determined at 450 nm using a microplate reader. The concentrations of these factors were calculated from standard curves established using standard samples.

### Measurement of Oxidative Stress Markers

The kidney tissues were homogenized in RIPA buffer, centrifuged at 12,000 rpm for 10 min at 4°C, and the supernatants were collected. Oxidative stress was evaluated by measuring the levels of glutathione peroxidase (GSH-PX), superoxide dismutase (SOD), and malondialdehyde (MDA). We determined the concentration of GSH-PX and MDA using the colorimetric method and thiobarbituric acid method, respectively, and measured the activity of SOD by the xanthine oxidase method.

### Western Blot

Renal tissues were ultrasonically homogenized in RIPA buffer (Servicebio, Wuhan, China) including a protease inhibitor cocktail and phosphatase inhibitor cocktail. The samples were centrifuged at 1,200 rpm for 10 min at 4°C, and the supernatants were collected as total proteins. Nuclear and cytoplasmic proteins, were extracted using the Nuclear and Cytoplasmic Protein Extraction Kits (Servicebio, Wuhan, China). Protein concentrations were measured using a BCA protein assay kit to ensure equal protein loading. Equal samples were loaded on polyacrylamide gel, electrophoresed, and transferred onto a polyvinylidene difluoride membrane. The membranes were blocked with 5% non-fat milk for 1 h and then incubated overnight at 4°C with primary antibodies: Bcl-2 antibody (GB13458, 1:750, Servicebio), Bax antibody (GB11690, 1:750, Servicebio), cleaved caspase-3 antibody (66470-2-lg, 1:750, Servicebio), Nrf2 antibody (AF7006, 1:1000, Affinity Biosciences), and HO-1 antibody (GB12104, 1:750, Servicebio). The next day, after washing with 1X Tris-buffered saline containing Tween (TBST), the membranes were incubated with a secondary antibody. In addition, the blots were probed for the expression of β-actin, which served as an internal control. After washing with 1X TBST, the bands were visualized by incubation with a luminescence reagent. ImageJ software was used for quantification.

### Statistical Analysis

Here, data were analyzed by the SPSS (version 22.0). All measurement data are presented as mean ± standard deviation (SD). One-way repeated-measure analysis of variance (ANOVA) was applied to perform omnibus comparisons among three or more groups. Student's *t*-test was used to test pairwise differences among the groups. Kaplan-Meier survival analysis was conducted to compare overall survival among the different groups. Statistical significance was set at *P* < 0.05.

## Results

### Baseline Physiological Parameters

**Figure 1:**
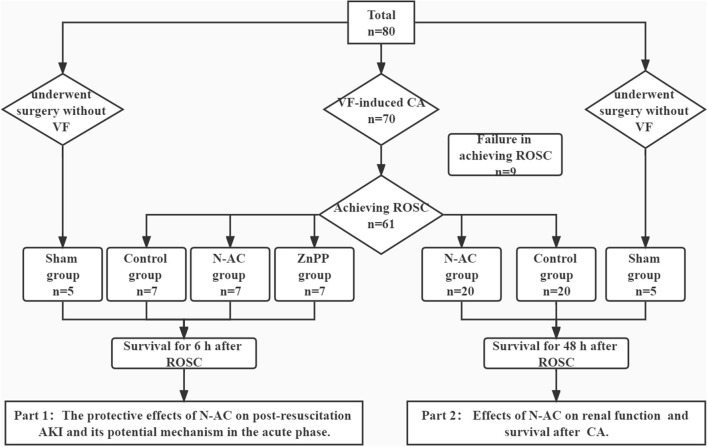
Flow diagram of the study. The whole study is consisted of 2 parts. VF, ventricular fibrillation; CA, cardiac arrest. N-AC, N- N-acetylcysteine; ROSC, return of spontaneous circulation; AKI, acute kidney injury.

In this study, 71 rats were survival after surgery: 10, 27, 27, and 7 rats in the sham, control, N-AC, and ZnPP groups, respectively (Figure 1). Additionally, resuscitation failed in nine rats. There were no statistically significant differences (*P* > 0.05) in baseline physiological measurements among the groups (Table 1).

**Table 1:** Baseline physiological parameters in groups.

**Group**	**Body weight [g]**	**Heart rate [bpm]**	**MAP [mmHg]**	**EF [%]**	**Temperature [°C]**	**pH**
SHAM	505 ± 11	386 ± 13	124 ± 4	74.8 ± 4.3	36.5 ± 0.4	7.43 ± 0.06
CONT	511 ± 15	383 ± 15	125 ± 7	73.2 ± 4.5	36.6 ± 0.4	7.44 ± 0.07
N-AC	508 ± 14	384 ± 19	127 ± 6	75.5 ± 5.4	36.4 ± 0.5	7.46 ± 0.08
ZnPP	506 ± 15	398 ± 12	129 ± 5	75.0 ± 3.8	36.8 ± 0.4	7.47 ± 0.05

*SHAM, sham group; CONT, control group; N-AC, N-AC group; ZnPP, ZnPP group. MAP, Mean Arterial Pressure; pH, Potential of Hydrogen; EF, Ejection Fraction. Sham group, n = 10; Control group, n = 27; N-AC group, n = 27; ZnPP group, n = 7*.

### N-AC Alleviated Renal Dysfunction and Histopathological Changes After ROSC

**Figure 2:**
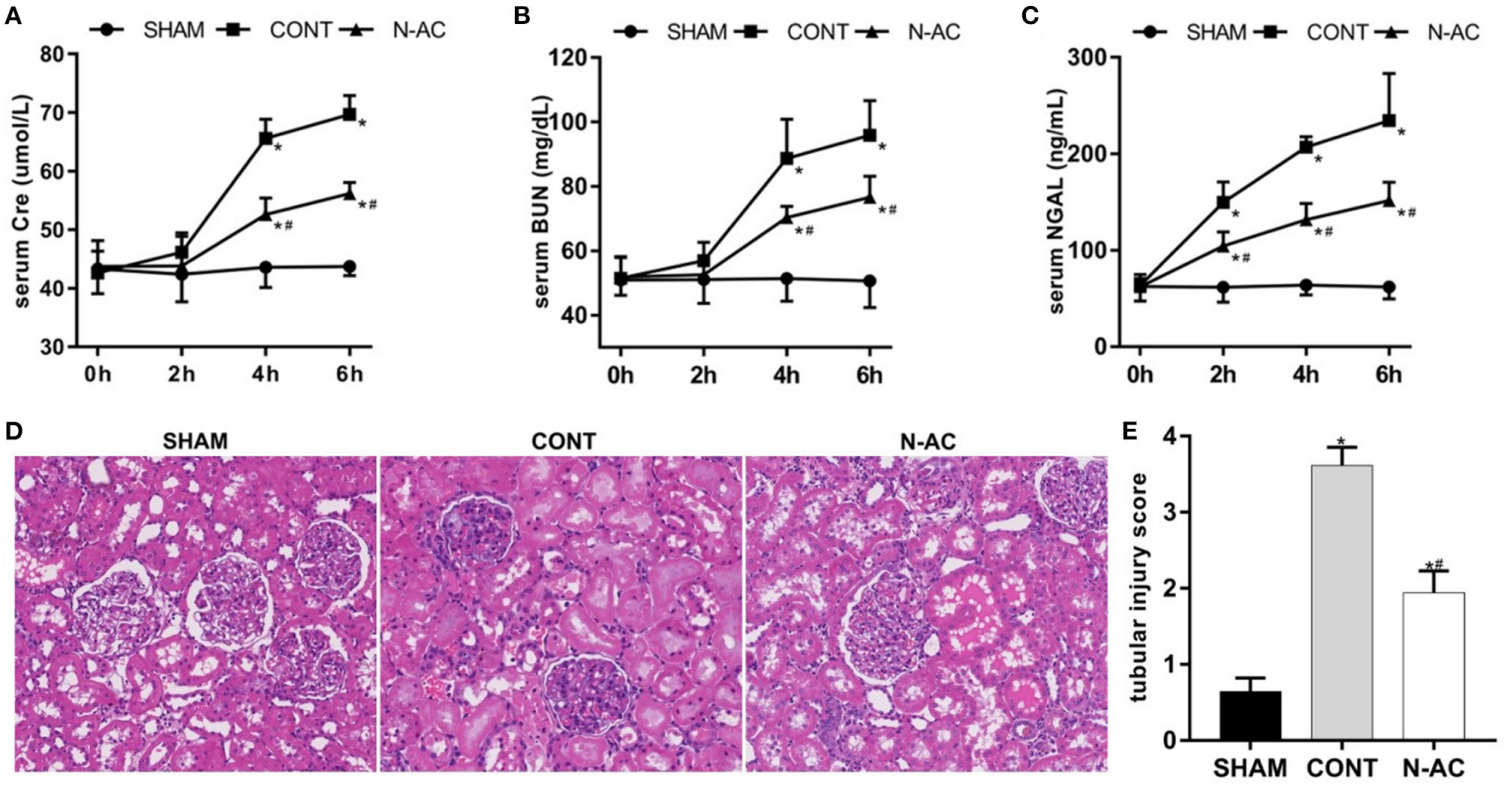
N-AC alleviated renal dysfunction and pathological changes at 6 h after ROSC. **(A)** The dynamic changes of serum creatinine (Scr) level after ROSC. **(B)** The dynamic changes of serum blood urea nitrogen (BUN) level after ROSC. **(C)** The dynamic changes of serum neutrophil gelatinase- associated lipocalin (NGAL) level after ROSC. **(D)** The renal pathological damage was evaluated by HE stains (400 × magnification). **(E)** Quantitative dates of TIS. SHAM, sham group; CONT, control group; N-AC, N-AC group. Sham group, *n* = 5; Control group, *n* = 7; N-AC group, *n* = 7. Data are expressed as mean ± SD. * *P* < 0.01, vs. sham group; # *P* < 0.01, vs. control group. SHAM, Sham group; CONT, Control group; N-AC, N-AC group.

A rat model of cardiac arrest was established to explore the effects of N-AC on post-resuscitation AKI. As shown in Figure 2, the biomarker levels of renal injury in control group, including SCr, BUN, and NGAL (Figures 2A–C, respectively), were apparently increased compared with those in the sham group. However, there were no marked changes in SCr and BUN levels at 2 h after ROSC. The levels of SCr and BUN were remarkably increased (*P* <0.01) at 4 h after ROSC. As a sensitive biomarker, the serum level of NGAL showed a significant increase at 2 h after ROSC (*P* < 0.01). This indicates that NGAL is of significant importance as an effective early predictor of post-resuscitation AKI. After treatment with N-AC, the increasing trends of SCr, BUN, and NGAL were effectively delayed and the serum levels of these biomarkers were significantly reduced compared to control group (*P* < 0.01). These results demonstrated that N-AC inhibited the increase in renal function parameters to some extent in rat CA models. We then assessed renal pathological changes induced by CA using HE staining. In the sham group, the kidney histological structure showed no marked destruction and tubular structures remained normal under the microscope. At 6 h after ROSC, we observed tubular injury (Figure 2D) and evaluated the TIS (Figure 2E). In the control group, we observed apparent structural damage to the renal tubules, including increases in vacuoles and lumen dilatation, brush border detachment, inflammatory cell infiltration, and intraluminal cast formation. Additionally, compared with the sham group, the TIS of the control group increased markedly. In the N-AC group, the severity of the pathological injury was alleviated to some extent. We also found that the renal tubular structure was relatively intact, and the TIS was reduced after treatment with N-AC.

### N-AC Inhibited ROS Generation in the Kidney After ROSC

**Figure 3:**
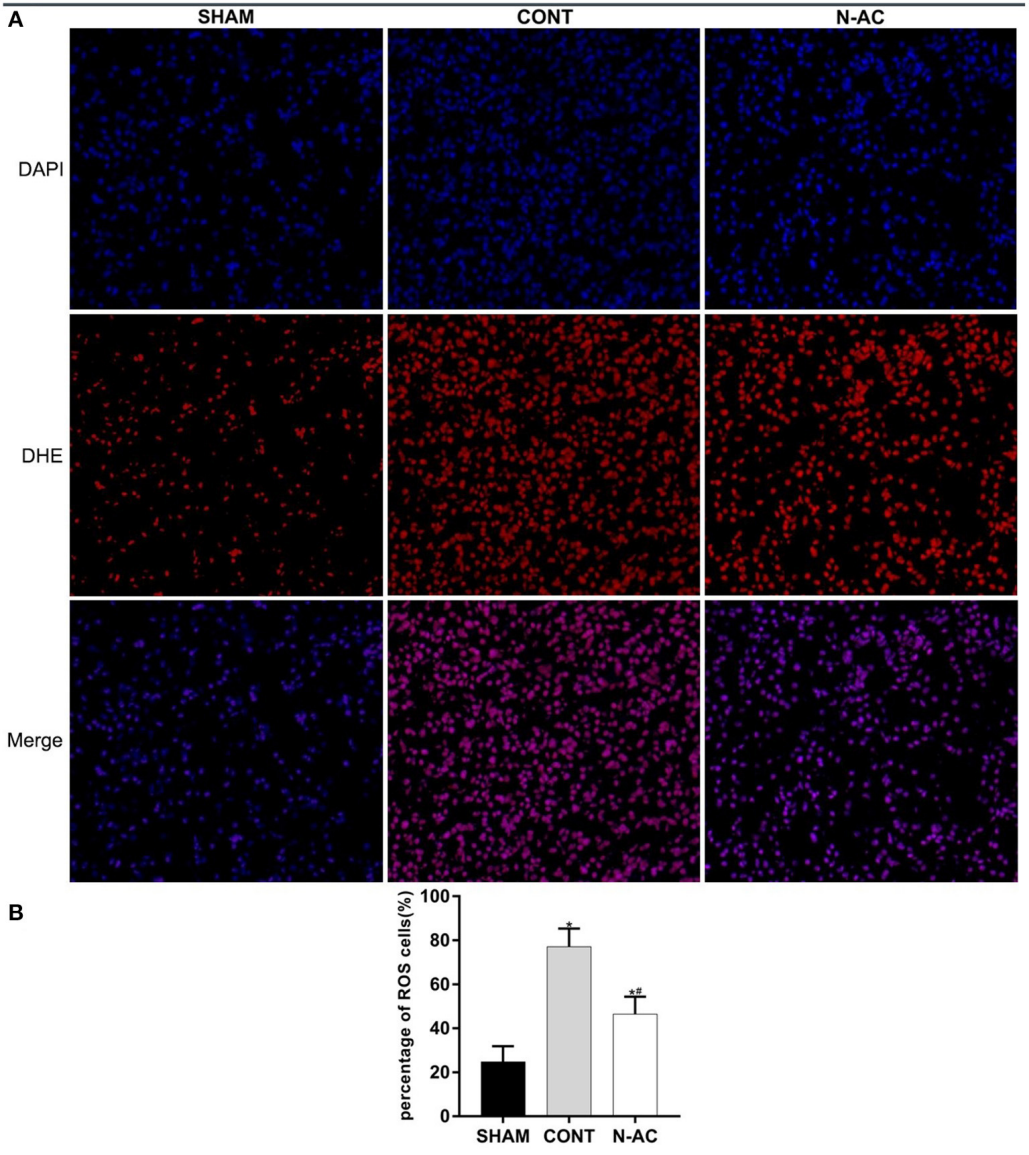
N-AC reduced the generation of ROS in the kidney at 6 h after ROSC. **(A)** Representative DHE staining showing the ROS levels in the kidney at 6 h after ROSC. Scale bars = 10 μm. **(B)** Percentage of ROS-positive cells defined as ROS-positive cells/DAPI-positive cells based on the DHE staining results. SHAM, sham group; CONT, control group; N-AC, N-AC group. Sham group, *n* = 5; Control group, *n* = 7; N-AC group, *n* = 7. Data are expressed as mean ± SD. * *P* < 0.01, vs. sham group; # *P* < 0.01, vs. control group.

ROS levels were examined by DHE staining and evaluated by fluorescence microscopy. At 6 h after ROSC, a considerable amount of ROS were observed in the control group (*p* < 0.01). After treatment with N-AC, ROS generation was significantly reduced (*p* < 0.01) (Figure 3).

### N-AC Reduced the Levels of Oxidative Stress in the Kidney After ROSC

**Figure 4:**
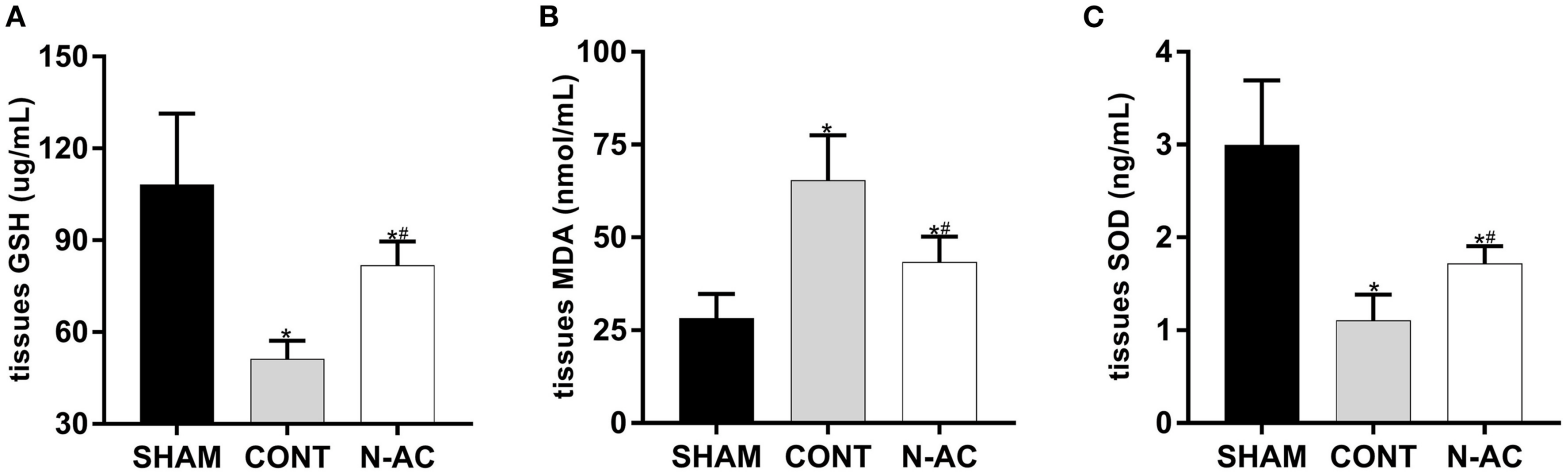
N-AC suppressed renal oxidative stress at 6 h after ROSC. **(A)** The concentration of GSH-PX in the renal tissues. **(B)** The concentration of MDA in the renal tissues. **(C)** The concentration of SOD in the renal tissues. SHAM, sham group; CONT, control group; N-AC, N-AC group. Sham group, *n* = 5; Control group, *n* = 7; N-AC group, *n* = 7. Data are expressed as mean ± SD. * *P* < 0.01, vs. sham group; # *P* < 0.01, vs. control group.

To demonstrate the effect of N-AC on oxidative stress after CPR, we determined the levels of GSH (Figure 4A), MDA (Figure 4B), and SOD (Figure 4C) in renal tissues. The concentrations of these indicators changed significantly as the principal parameters of oxidative stress. The levels of antioxidants (GSH-PX and SOD) in control group were markedly decrease compare with sham group at 6 h after ROSC, while the content of peroxide (MDA) was markedly increased. Treatment with N-AC significantly decreased the concentration of MDA and apparently increased the concentrations of SOD and GSH-PX in the N-AC group. These results suggested that oxidative stress responses were apparent in the kidney after CPR, and N-AC significantly inhibited renal oxidative stress in cardiac arrest models (Figure 4).

### N-AC Attenuated Cell Apoptosis in the Kidney After ROSC

**Figure 5:**
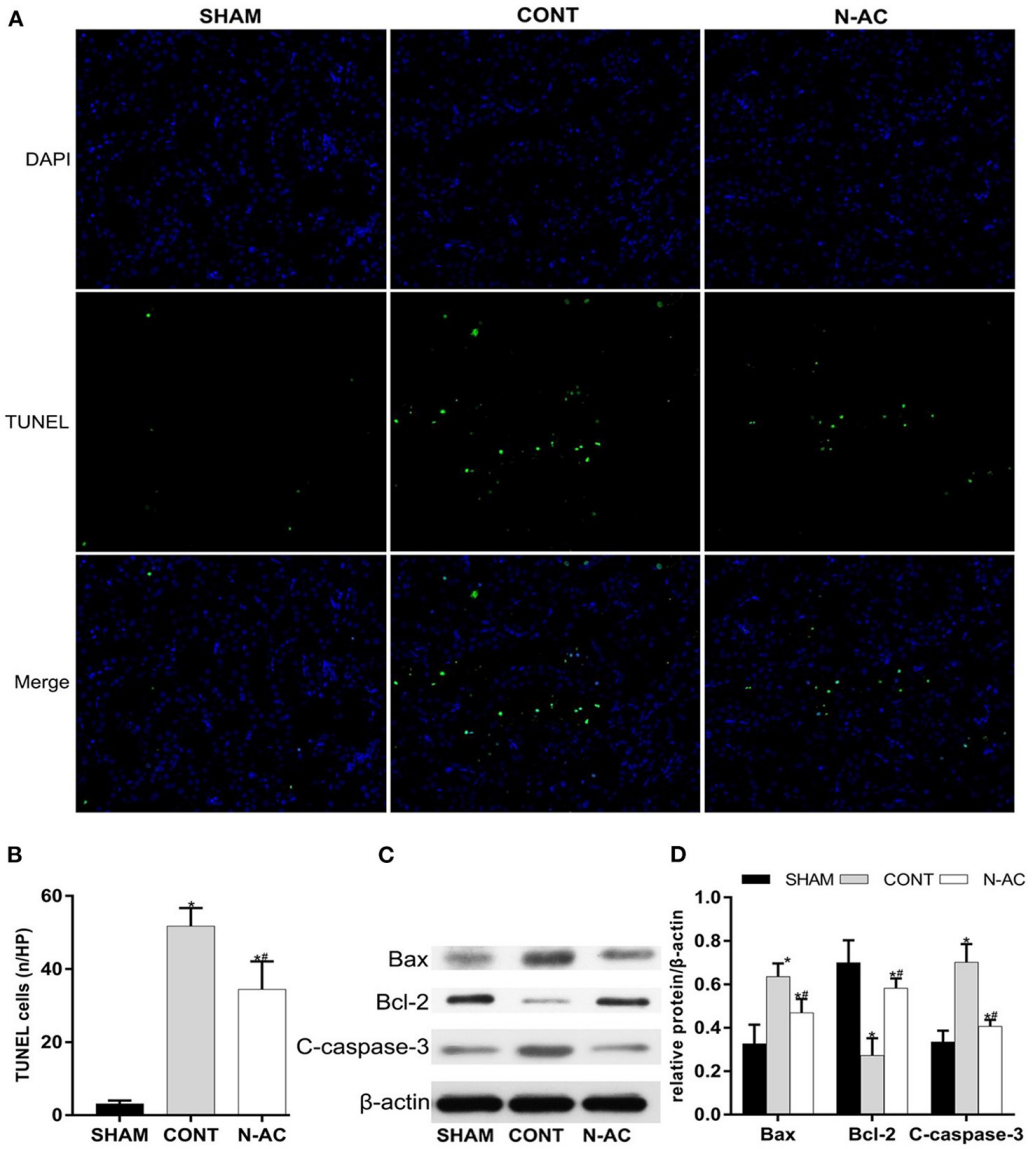
N-AC inhibited renal cell apoptosis in the CPR models at 6 h after ROSC. **(A)** TUNEL staining is used to detect the numbers of apoptotic cells in the kidney. **(B)** The rate of TUNEL-positive cells in three groups. **(C)** The protein levels of Bax, cleaved caspase-3 and Bcl-2 was evaluated via western blot. **(D)** The densitometric analysis of the expression of Bax, Bcl-2 and cleaved caspase-3. SHAM, sham group; CONT, control group; N-AC, N-AC group. Sham group, *n* = 5; Control group, *n* = 7; N-AC group, *n* = 7. Data are expressed as mean ± SD. *P < 0.01, vs. sham group; #*P* < 0.01, vs. control group. C-caspase-3, cleaved caspase-3.

TUNEL staining was performed to measure renal cell apoptosis in the kidney. The control group had a higher number of TUNEL-positive cells than the sham group did (Figure 5A). Additionally, the number of positive cells in the N-AC group was markedly lower than that in the control group (Figure 5B). Meanwhile, the protein expression of apoptosis-related factors, including cleaved caspase-3, pro-apoptotic factors Bax, and anti-apoptotic factor Bcl-2, was determined by western blotting (Figure 5C). At 6 h after ROSC, Bax protein expression was increased and Bcl-2 expression was decreased in the control group. Contrastingly, N-AC inhibited Bax increase and Bcl-2 decrease (Figure 5D). Cleaved caspase-3, a crucial factor in the process of cell apoptosis, was apparently increased in the control group. N-AC effectively reversed this change. Based on the findings of the TUNEL assay and western blotting, we confirmed that N-AC suppressed renal cell apoptosis in CPR models (Figure 5).

### N-AC Further Upregulated the Nuclear Translocation of Nrf2 and the Expression of HO-1 in the Kidney After ROSC

**Figure 6:**
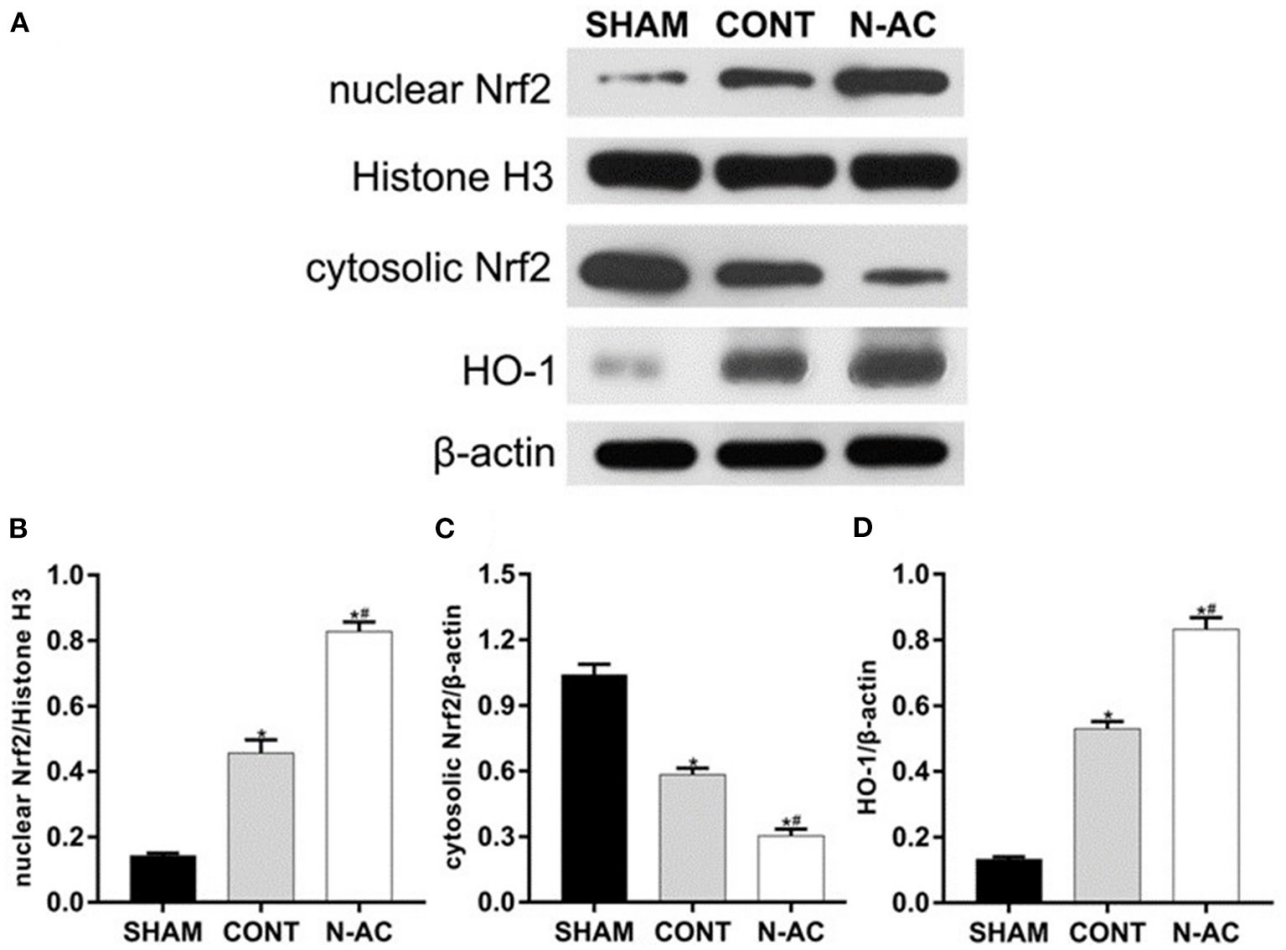
N-AC enhances nuclear Nrf2 accumulation and upregulates the expression of HO-1 in the kidney at 6 h after ROSC. **(A)** Representative images of nuclear Nrf2, cytosolic Nrf2, and HO-1 protein levels in each group at 6 h after ROSC. **(B)** The densitometric analysis of nuclear Nrf2 expression relative to the loading control. **(C)** The densitometric analysis of cytosolic Nrf2 expression relative to the loading control. **(D)** The densitometric analysis of HO-1 expression relative to the loading control. SHAM, sham group; CONT, control group; N-AC, N-AC group. Sham group, *n* = 5; Control group, *n* = 7; N-AC group, *n* = 7. Data are expressed as mean ± SD. *P < 0.01, vs. sham group; #*P* < 0.01, vs. control group.

To demonstrate N-AC molecular mechanism of action on post-resuscitation AKI, we extracted and isolated nuclear and cytoplasmic proteins. We detected the protein expression of Nrf2 and HO-1 in the cell nucleus and cytoplasm, respectively by western blotting (Figure 6A). Surprisingly, nuclear Nrf2 expression in the control and N-AC groups increased (Figure 6B). This result indicated that nuclear translocation of Nrf2 was increased in the kidney after CPR. Moreover, N-AC treatment further increased the accumulation of nuclear Nrf2 in the N-AC group compared with the control group (Figure 6B). We hypothesized that the former may be associated with the activation of the antioxidative response, and the latter may be attributed to the enhancement of antioxidative ability mediated by N-AC treatment. In the sham group, the basal level of HO-1 was low (Figure 6D). However, at 6 h after ROSC, HO-1 expression increased in the kidney tissues (Figure 6D). Moreover, with the intervention of N-AC, HO-1 protein expression was apparently increased in the N-AC group compared with the control group (Figure 6D). These results indicated that N-AC further promoted Nrf2 nuclear translocation of Nrf2 and upregulated HO-1 expression (Figure 6).

### Inhibition of HO-1 Activity Partly Abolished N-AC-Mediated Renal Protective Effects After ROSC

**Figure 7:**
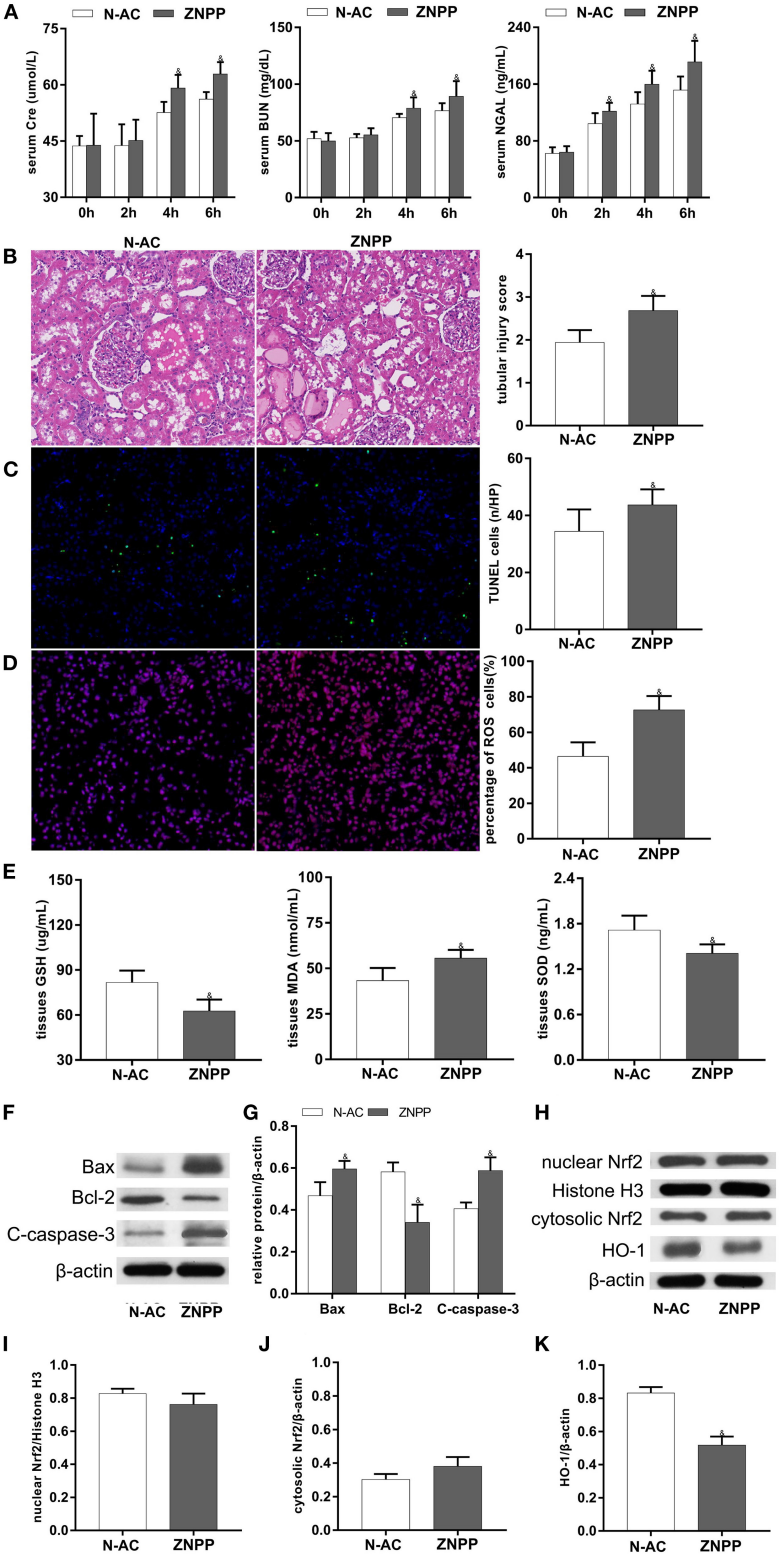
The participation of HO-1 in the protective effects of N-AC after CPR in the kidney was clarified by using the specific HO-1 inhibitor, ZnPP. **(A)** The dynamic changes of Scr, BUN and NGAL after ROSC. **(B)** HE staining determines pathological changes in the kidney. **(C)** TUNEL staining detects the numbers of apoptotic cells. **(D)** Representative DHE staining showed the ROS levels in the kidney. **(E)** The contents of SOD, GSH-PX and MDA in the kidney tissues. **(F)** Protein expression of Bax, cleaved caspase-3, Bcl-2. **(G)** The densitometric analysis of apoptotic proteins. **(H)** The expressions of Nrf2 in cell nucleus and cytoplasm and the expression of HO-1 in whole cell were detected using Western blot. **(I)** The densitometric analysis of nuclear Nrf2 expression relative to the loading control. **(J)** The densitometric analysis of cytosolic Nrf2 expression relative to the loading control. **(K)** The densitometric analysis of HO-1 expression relative to the loading control. N-AC, N-AC group; ZnPP, Znpp group. N-AC group, *n* = 7; ZnPP group, *n* = 7. Data are expressed as mean ± SD. & *P* < 0.05, vs. N-AC group. ZNPP, ZnPP group.

ZnPP, a specific HO-1 inhibitor, was used to further demonstrate that N-AC protective effects on post-resuscitation AKI were mediated through Nrf2/HO-1 pathway activation. Compared with the N-AC group, pretreatment with ZnPP in the ZnPP group resulted in decreased expression of HO-1 (Figure 7H), increased serum levels of renal function biomarkers (Figure 7A) and worsened renal injury on histopathological examination (Figure 7B). Furthermore, cell apoptosis (Figures 7C,F), and oxidative stress (Figure 7E) were enhanced, and ROS production (Figure 7D) was significantly increased. The above results demonstrated that inhibition of HO-1 activity markedly reversed N-AC-induced protective actions against renal injuries, and these protective effects were mediated, in part, by Nrf2/HO-1 antioxidative axis activation (Figure 7).

### N-AC Improved Post-resuscitation AKI and Survival Outcome at 48 h After ROSC

**Figure 8:**
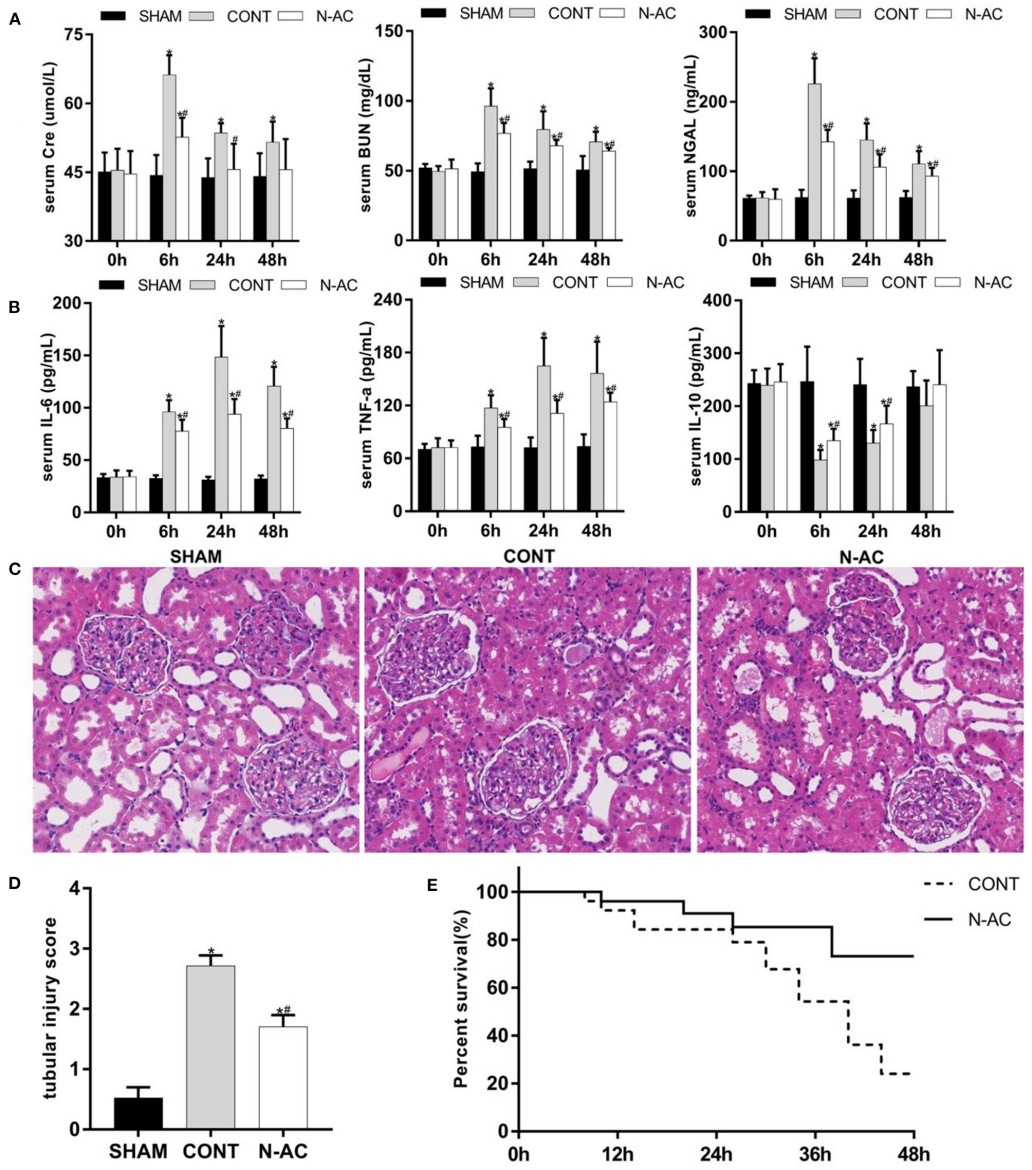
N-AC exert renal protective effects and improve survival rate at 48 h after ROSC. **(A)** The dynamic changes of renal function in each group. **(B)** The dynamic changes of inflammatory response in each group. **(C)** Pathological changes in the kidneys. **(D)** Quantitative dates of TIS. **(E)** The Kaplan-Meier survival curves of control group and N-AC group. SHAM, sham group; CONT, control group; N-AC, N-AC group. Sham group, *n* = 5; Control group, *n* = 20; N-AC group, *n* = 20. Data are expressed as mean ± SD. * *P* < 0.05, vs. sham group; # *P* < 0.05, vs. control group.

We examined relative indicators that reflect the magnitude of renal injury at 48 h after ROSC to determine whether N-AC exerted the same effects in the long-term survival model. In the control group, the derangement of renal function indices at 48 h after ROSC in relation to that at ROSC 6 h was milder. However, there was a statistically significant difference when renal function indices were compared to that of the sham group. In the N-AC group, the renal function significantly improved after treatment with N-AC compared with the control group (Figure 8A). Similar results were observed in the inflammatory response (Figure 8B). Although the inflammatory response gradually intensified and reached a peak at 24 h after ROSC, the serum levels of pro-inflammatory cytokines (IL-6 and TNF-α) were markedly decreased and that of anti-inflammatory cytokine (IL-10) was markedly increased after treatment with N-AC at 48 h after ROSC. Surprisingly, the serum level of IL-10 was increased at 48 h after ROSC in control group. Concurrently, the results of HE staining further indicated that N-AC alleviated renal injury at 48 h after ROSC (Figure 8C). More importantly, Kaplan-Meier survival analysis revealed that administration with N-AC resulted in a substantial improvement in survival. At 48 h after ROSC, the survival rates in control rats were 40%, however in the N-AC group, survival rate was significantly improved and could rise to 80% (Figure 8E). These results indicated that the administration of N-AC reduced renal damage and significantly improved outcomes in the CPR model (Figure 8).

## Discussion

In this study, we investigated the protective effects of N-AC and its potential mechanism of action via the activation of the Nrf2/HO-1 pathway in post-resuscitation AKI. Our results indicated that post-resuscitation AKI was significant and presented with progressive aggravation, accompanied by the release of a large amount of ROS and inflammatory cytokines and the enhancement of oxidative stress and cell apoptosis. Additionally, our results demonstrated that N-AC has protective effects on post-resuscitation AKI and that this action is partly mediated by the activation of Nrf2/HO-1 pathway. This was supported by the fact that inhibition of HO-1 expression by ZnPP reversed the protective effects of N-AC on post-resuscitation AKI. Most importantly, N-AC significantly prolonged the duration of survival in CPR models.

Whole-body ischaemia due to cardiac arrest and subsequent CPR together contribute to PCAS, which is a multiple organ dysfunction syndrome, resulting in extremely high mortality. The latest epidemiological data, reported by the International Liaison Committee on Resuscitation, indicated that the in-hospital mortality rate of PCAS patients was up−79.4–96.9% during the first 30 days after CPR (5). Due to the importance of the brain and heart, researchers in PCAS predominantly focused on post-resuscitation myocardial dysfunction and brain injury, and studies on AKI are limited. However, post-resuscitation AKI is highly prevalent in CA patients, with a reported incidence of up to 48% (6). Moreover, injured kidneys play a vital role in the progression of PCAS. Post-resuscitation AKI could lead electrolyte and acid-base disturbances, such as hyperkalaemia and metabolic acidosis, which are associated with adverse cardiovascular events. It could also progress to acute renal failure such that patients with PCAS require renal replacement therapy, and such patients may develop chronic kidney disease (CKD) in the long-term. Despite the increasing success rate of ROSC, the incidence and mortality of post-resuscitation AKI remain significant (31, 32). Some substances were proven to partly alleviate post-CPR AKI to some extent in animal model (33, 34); however, there is insufficient evidence to demonstrate that their pharmacological effects can improve long-term survival. Therefore, there is an urgent need to explore novel and feasible therapeutic strategies to prevent AKI and improve outcomes after CPR.

Cardiac arrest obviously induces global ischaemic injury, but reperfusion injury also occurs after ROSC. I/R injury during resuscitation is a major contributor to post-resuscitation AKI. Only a low amount of ROS is generated during ischaemia, while large quantities of ROS are produced during reperfusion. With substantial ROS accumulation, the balance between oxidative and antioxidative responses in the kidney is disturbed. Further, the imbalance of the redox state induces oxidative stress and causes the peroxidation of lipids, proteins, and DNA, which in turn damages the function and structure of renal tubular cells (35). Even worse, ROS induce tissue injury in multiple ways. Mitochondria are known to be the major site for the generation of ROS, as well as the sensitive place of ROS. Excessive ROS causes mitochondrial oxidative stress and impairs mitochondrial function (36). ROS-mediated oxidative stress and endoplasmic reticulum (ER) stress also result in AKI (37). Additionally, a large amount of ROS promotes inflammatory response through multiple pathways (38, 39). Moreover, mitochondrial dysfunction, oxidative stress, ER stress, and inflammatory responses mediated by ROS promote cell apoptosis (40). Worse still, the production of ROS appears to be the main element in the progression of AKI to CKD and end-stage renal disease (41). These ROS-associated mechanisms of injury eventually cause AKI. In summary, the excessive ROS contribute to further damage ischemic kidney and play a critical role in the development of post-resuscitation AKI. Therefore, inhibiting the formation of ROS in I/R may be a valid therapeutic strategy of post-resuscitation AKI.

In the present study, our results demonstrated the overproduction of ROS and the activation of the ROS-mediated damage pathway in the CPR model. After CPR, oxidative stress, inflammatory response, and cell apoptosis apparently increased with the accumulation of ROS. N-AC, a specific antidote for acetaminophen poisoning, has been reported to exhibit ROS-scavenging properties (42). In a variety of animal models, N-AC has been shown to exert protective effects by reducing ROS. He et al. reported that N-AC can improve myocardial dysfunction by decreasing oxidative stress and inhibiting ROS-mediated NLRP3 inflammasome-induced pyroptosis (24). Hong et al. reported that N-AC can prevent hyperglycaemia-induced mesangial apoptosis and fibrogenesis by reducing ROS to inhibit the formation of MDA (43). Additionally, N-AC has been reported to protect against I/R injury. For example, previous research revealed that N-AC improved renal function by improving hemodynamics and inhibiting ROS-mediated lipid hydroperoxides (26). In our study, we confirmed that N-AC effectively improved renal function and alleviated the pathological damage of renal tissue. Our results provide evidence that the protective effects of N-AC against post-resuscitation AKI may be closely associated with the inhibition of ROS-induced oxidative stress, inflammation, and cell apoptosis. However, the therapeutic effects of N-AC disappeared after the administration of ZnPP, a specific inhibitor of HO-1.

Haem oxygenase (HO) is a rate-limiting enzyme, which catalyzes the degradation of haem, producing carbon monoxide (CO), bilirubin, and iron. To date, three isoforms of HO have been reported in mammals, including the inducible form HO-1 and two constitutive forms HO-2 and HO-3, while a fourth isoform (HO-4) has been found in plants (44, 45). Under physiological conditions, the expression level of HO-1 is low in most tissues. Under various conditions, including I/R, inflammation, and oxidative damage, HO-1 is adaptively activated within hours in response to multiple cellular stress inducers (46). However, deletion of the HO-1 gene leads to aggravation of tissue injury (47). Consequently, HO-1 is considered a cytoprotective enzyme. Increasing its expression level and activity could play a cytoprotective role in defending against oxidative injury and other types of damage. The expression of HO-1 is regulated mainly at the transcription level. The basal and inducible expression of HO-1 is mediated through the cis-regulatory antioxidant response element (ARE) sequence located in the promoter region. The induction procedure involves the structural activation of transcription factors and their translocation into the nucleus (48). Nuclear accumulation of Nrf2, a transcription factor, induces the activation of HO-1 at the transcriptional level. Under physiological conditions, Nrf2 is rendered inactive by its cytoplasmic retention by the inhibitor Keap 1 which targets Nrf2 for proteasomal degradation. When exposed to endogenous factors such as electrophiles or ROS, Nrf2 dissociates from Keap1 and translocates into the nucleus. In the nucleus, the activated form of Nrf2 accumulates and binds to ARE, upregulating the expression of cytoprotective genes, including HO-1 (49). As suggested by our results, ROS generation was significantly increased in the kidneys after CPR. Nrf2 accumulated in the nucleus, and endogenous HO-1 was increased in the kidney after CPR. The activation of this adaptive response could enhance cellular resistance. However, this adaptive response was not sufficient to protect the kidneys from injury after CPR. Recently, it has been suggested that Nrf2/HO-1 pathway was activated in the early stage after ROSC. However, the expression of Nrf2/HO-1 decreased, the renal injury and dysfunction increased, and the survival rate of rats decreased during later stage after ROSC (50). Therefore, the activation of Nrf2/HO-1 pathway may be a promising therapeutic target on post-resuscitation AKI. Treatment with N-AC significantly enhanced the activation of the Nrf2/HO-1 antioxidative pathway after CPR and eventually protected the kidneys from I/R injury. Apart from N-AC, there are a variety of other renal protective substances, including phosphocreatine (51), Ginkgo biloba extract (52), and Embelin (53). These agents have also been demonstrated to possess beneficial effects in the treatment of different renal diseases via activation of the Nrf2/HO-1 antioxidative pathway. Using transgenic knockout mice, researchers validated that the activation of HO-1 was involved in cilostazol-induced hepatoprotective effects against hepatic I/R injury (54). In the I/R model, the HO-1 activator cobalt protoporphyrin (CoPP) has been proven to be effective in attenuating I/R-induced damage to multiple organs (55, 56). In contrast, administration of the HO-1 inhibitor ZnPP abolished the therapeutic effects of pterostilbene on I/R-induced cerebral damage (57). The protective actions of HO-1 have been attributed to its enzymatic products, including bilirubin, CO, and iron. HO-1 metabolizes biliverdin to bilirubin; both biliverdin and bilirubin are endogenous cytoprotective antioxidants that efficiently scavenge ROS, inhibit lipid peroxidation, and increase antioxidant enzymes (58). As shown in this study, N-AC administration resulted in a great reduction in the generation of ROS and MDA and simultaneously enhanced SOD and GSH levels. In contrast, CO is a signal molecule that possesses anti-apoptotic and anti-inflammatory properties. Furthermore, drug-related upregulation of HO-1 expression attenuated I/R injury by inhibiting cell apoptosis (59) and reducing the expression of inflammatory genes (60). This was confirmed by our experimental results. With the upregulation of HO-1 expression by N-AC induction, TNF-α and IL-6 were decreased and the IL-10 was increased. This indicated that N-AC was able to enhance HO-1 mediated anti-inflammatory responses. The anti-apoptotic activity of HO-1 in the kidney was also improved. Overall, we confirmed that N-AC could enhance the renal adaptive antioxidative ability. We also demonstrated that modulation of the Nrf-2/HO-1 antioxidative axis contributed to the protective properties of N-AC in post-resuscitation AKI.

In order to verify the role of N-AC on improving outcomes in a CPR model, we preformed 48 h survival experiments. At 48 h after ROSC, the continuous deterioration of biochemical indicators of renal function were partly delayed with reperfusion of systemic blood flow in the control group, but this improvement was not representative of the alleviation of post-resuscitation AKI. Meanwhile, inflammatory response gradually intensified over time and reached a peak at 24 h, but it is surprised that the serum level of IL-10 rebounded at 48 h after ROSC. This may be associated with endogenous stress response, which activated anti-inflammatory response to resist the over activated pro-inflammatory response. More importantly, our results revealed that post-resuscitation AKI and systemic inflammation could be effectively relieved after treatment with N-AC, and the outcomes of animal models of CA were also markedly improved. Moreover, in multiple animal models, researchers have demonstrated the protective effects of N-AC. In a stroke model, researchers found that N-AC attenuated cerebral ischemia and reperfusion injury (60). In addition, He et al. demonstrated that N-AC improved myocardial dysfunction by inhibiting ROS in a CA model (24). Based on our findings and findings from other studies, we believe that the improvement in survival is probably related to the multiorgan-protective effects of N-AC in the CPR model, and that N-AC plays a crucial role in protection against post-resuscitation AKI. However, whether the multiorgan protective effects of N-AC are related to activation of the Nrf2/HO-1 antioxidative pathway remains to be further investigated.

The study had several limitations. First, we did not carry out the corresponding pilot studies to select the optimal concentration of N-AC. However, after reviewing a large amount of literature, we determined the mode of administration and concentration. N-AC was administered through the femoral vein after ROSC at a dose of 150 mg kg-1. Second, we did not demonstrate the mechanisms of action of N-AC in a vitro hypoxia/reoxygenation model. *In vitro* model may not simulate the complex processes of CPR model. Therefore, we only carried out experiments on an animal model and used the HO-1 inhibitor ZnPP to clarify the protective mechanism of N-AC in the kidney. Nonetheless, *in vitro* experiments would be taken in to consideration in future studies. Third, at 48 h after restoration of ROSC, we conducted survival research and evaluated the protective effects of N-AC on the kidney after CPR. However, we did not clarify the variation in the Nrf2/HO-1 antioxidative pathway. Further studies to fully investigate the protective mechanisms of N-AC is needed, and we believe that these studies will address the issues raised above.

## Conclusion

In this study, we demonstrated that Nrf2/HO-1 pathway is involved in the pathologic process of post-resuscitation AKI. N-AC alleviates post-resuscitation AKI by activating the Nrf2/HO-1 antioxidative pathway and efficiently prolongs the survival duration in rat models of CPR. Taken together, these findings suggest that HO-1 is expected to become a new target for the treatment of post-resuscitation AKI, and N-AC hold pharmaceutical potential in treating CPR-related renal diseases.

## Data Availability Statement

The original contributions presented in the study are included in the article/supplementary material, further inquiries can be directed to the corresponding author/s.

## Ethics Statement

The animal study was reviewed and approved by the Animal Care and Use Committee of Ren Ji Hospital, School of Medicine, Shanghai Jiao Tong University.

## Author Contributions

CZ, XL, and CW contributed to the conception of the study and supervised the manuscript. SW, GL, and TJ performed the experiment. SW and GL analyzed most of the data and wrote the initial draft of the paper. LT and QY contributed to consulting corresponding literatures, refining the ideas, and finalizing this paper. All authors read and approved the final manuscript.

## Funding

This work was supported by the National Natural Science Foundation of China (grant numbers 81971803 and 81671881).

## Conflict of Interest

The authors declare that the research was conducted in the absence of any commercial or financial relationships that could be construed as a potential conflict of interest.

## Publisher's Note

All claims expressed in this article are solely those of the authors and do not necessarily represent those of their affiliated organizations, or those of the publisher, the editors and the reviewers. Any product that may be evaluated in this article, or claim that may be made by its manufacturer, is not guaranteed or endorsed by the publisher.
